# A Differential Evolution-Based Routing Algorithm for Environmental Monitoring Wireless Sensor Networks

**DOI:** 10.3390/s100605425

**Published:** 2010-06-01

**Authors:** Xiaofang Li, Lizhong Xu, Huibin Wang, Jie Song, Simon X. Yang

**Affiliations:** 1 College of Computer and Information, Hohai University, Nanjing, China; E-Mails: lzhxu@hhu.edu.cn (L.Z.X.); hbwang@hhu.edu.cn (H.B.W.); 76397384@qq.com (J.S.); 2 School of Engineering, University of Guelph, Guelph, Canada; E-Mail: syang@uoguelph.ca

**Keywords:** environmental monitoring, meteorological and hydrological telemetry, Wireless Sensor Networks, Differential Evolution Algorithm, LEACH protocol

## Abstract

The traditional Low Energy Adaptive Cluster Hierarchy (LEACH) routing protocol is a clustering-based protocol. The uneven selection of cluster heads results in premature death of cluster heads and premature blind nodes inside the clusters, thus reducing the overall lifetime of the network. With a full consideration of information on energy and distance distribution of neighboring nodes inside the clusters, this paper proposes a new routing algorithm based on differential evolution (DE) to improve the LEACH routing protocol. To meet the requirements of monitoring applications in outdoor environments such as the meteorological, hydrological and wetland ecological environments, the proposed algorithm uses the simple and fast search features of DE to optimize the multi-objective selection of cluster heads and prevent blind nodes for improved energy efficiency and system stability. Simulation results show that the proposed new LEACH routing algorithm has better performance, effectively extends the working lifetime of the system, and improves the quality of the wireless sensor networks.

## 1. Introduction

Monitoring applications using a single detection device can only obtain local information. If a number of distributed sensors are used to collect data for broader monitored regions or for monitoring tasks with higher precision requirements, and to send those data and perhaps partially processed results to users via mutual collaboration and communication, the information collected by distributed sensor network systems will be more accurate and comprehensive, and the system’s robustness will be stronger. This concept is an important driving force for the development of wireless sensor networks (WSNs).

WSNs are intelligent network application systems that autonomously collect, integrate and transmit data. As an emerging information acquisition technology integrating the latest technological achievements in micro-electronic, network and communications, WSNs have been widely used in many fields such as the military, environmental monitoring, industrial control, and urban transportation [1]. In comparison to traditional means of environmental monitoring, WSN technology has the following advantages: (1) sensor nodes are small in size and the whole network needs to be deployed only once, so the human impact of the deployment on the monitored environment is very small, which is especially important for environments very sensitive to alien biological activities. Research on the living environment of petrels on the Great Duck Island in the United States showed that even a few minutes of researcher activity on the island would result in a 20% increase in the death rate of fledging birds [2]; (2) sensor network nodes are large in number, with a high-density distribution, while each node can detect the local environmental information and submit the details to the monitoring center. Sensor networks feature high volume and precision of data acquisition; and (3) wireless sensor nodes have a certain degree of computing power and storage capacity by themselves and can take on more complex monitoring based on changes in the physical environment. Sensor nodes can also communicate wirelessly and monitor collaboratively between nodes.

WSN technology is based on, but different from, the traditional wireless *ad hoc* network. The energy of nodes in WSNs is limited, as is their computing, communication and storage capability [3]. Energy-saving is an essential prerequisite for their reliable operation, and effective energy and resource management has become an important part of the technical issues to be considered, and consequently research on energy consumption has attracted more and more attention [4].

**Figure 1.**
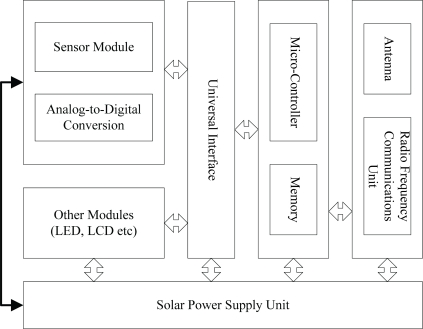
WSN Nodes for environmental monitoring.

The design of sensor network nodes used for environmental monitoring is shown in Figure 1. The design features dedicated universal interfaces for common sensors, which can be connected to five types of sensors: water level, precipitation, evaporation, flow and water quality. The circuit of each sensor is designed to be low-power, and its processor can be directed to sleep and wake-up via radio frequency instructions. Meanwhile at the same time sensors are equipped with solar cells to extend their lifetime. With the clustering routing protocol, hydrological information parameters collected within a certain period of time by sensors in a certain number within clusters will be converged to a selected cluster head node. After the fusion treatment, the information will be sent to a sink node and then to a monitoring center via some means of long-distance communication such as GPRS.

From the perspective of network resources, different protocol layers can use different strategies and techniques to meet the energy-saving goal. From the point of view of network layers, many routing protocols are designed and used to improve the network scalability and to extend its network life cycle for WSN applications, such as the energy-based routing protocol [5], the SPIN negotiation-based routing algorithm [6], the MTE minimum transmission energy protocol [7], and the data-centric directed diffusion protocol [8]. Among them, the routing protocol based on the clustering mechanism is used comparatively more than others [9].

In a monitored environment with a large-scale deployment of sensor nodes, the monitored properties (such as temperature, humidity, light, wind and precipitation) are usually distributed “structurally” with greater redundancy. Let us consider for example the precipitation distribution of the Taihu Lake Basin, the branch river system in the downstream region of the Yangzi river, centered at Taihu Lake with the drainage channel of the Huangpu river. The river system occupies an area of 36,500 km^2^, including Tianmu Mountain in Zhejiang Province and Maoshan Mountain in Jiangsu Province on the west, the end of Yangzi River on the north, facing the East China Sea and Hangzhou Bay on the east and south respectively. The precipitation in the west part of Zhejiang Province (Zhexi, in short) is higher than other areas no matter what year. The precipitation in Pudong and Puxi of Shanghai (a city located at the Coast of East Sea) and Yangcheng Dianmao area of Suzhou City is within the low value area of the precipitation line in every year. That is due to the impact of orographic lift on the precipitation. Zhexi is the only mountain area in the basin while Yangcheng Dianmao is a well-known low-lying land. The spatial distribution of the precipitation in the basin is that it is large in the former and small in the latter. This illustrates the impact of orographic lift on the precipitation. According to years of observation data, the general trend of the precipitation is that the upstream is larger than the downstream, the west than the east, the south than the north and the mountain than the plain in terms of space [10].

However, from the existing general pattern of precipitation information extraction, it can be seen that the system-level precipitation information processing technology is essentially a background approach of information processing that distributes measuring nodes based on geographical characteristics and processes data centrally. Although the information can be converged after all the data are sent separately to the background, the results obtained from this method are usually not as accurate as those obtained from the fusion in the front, and sometimes even produce fusion error. The participation of local information from the data source is generally required in information fusion, such as time and location of data was generated, and the location generating the data.

The clustering mechanism-based routing protocol [9] demonstrates its superiority. Its idea is that in a multi-hop communications process, a cluster head is used in data fusion to reduce the amount of data sent to sink nodes and to achieve the purpose of efficient use of energy. Compared with the flat routing protocols (flooding, gossiping, *etc.*), the introduction of the clustering mechanism improves performance significantly for similar or dissimilar sensor fusion of data collection with high correlation (or high similarity).

An earlier proposed one is the Low Energy Adaptive Clustering Hierarchy (LEACH) protocol [11,12] (referred as the traditional LEACH protocol in this paper). This idea of hierarchy-based protocol design has been accepted by more and more scholars. However there are still some problems in the practice of this routing protocol, the most important of which concerns the unreasonable selection of cluster heads, such as uneven distribution, no regard to the remaining energy of nodes, *etc.* Some improved algorithms have been proposed to solve these problems [13–15]. However, most of them select cluster heads according to the status information of candidate nodes without considering the information of their neighbors, which tends to result in blind nodes inside clusters, possibly reducing the survival time of the network.

This paper considers monitoring applications for an outdoor environment such as meteorological and hydrological data or the wetland ecology environment, and aims to reduce network energy consumption. It mainly focuses on the optimal selection of cluster heads, improves the traditional LEACH routing algorithm, and introduces and designs a routing algorithm based on the differential evolution algorithm (DE), the DE_LEACH routing algorithm in short. The DE_LEACH algorithm uses the easy and fast search features of the DE in multi-objective optimization applications, takes energy and distance distribution of neighbor nodes inside clusters into account, and then optimizes the selection of cluster heads. The algorithm has advantages in terms of effectively preventing premature blind nodes and reducing network energy consumption, and is more suitable than others to meet the needs of WSN applications in monitoring applications for outdoor environment such as those mentioned before.

The rest of this paper is organized as follows: Section 2 reviews related work on WSN protocols; Section 3 describes the idea of differential evolution algorithms; Section 4 presents the proposed method; Section 5 proves the DE_LEACH algorithm through experiments and analyzes the experiment results, and finally, Section 6 concludes and indicates several issues for future work.

## 2. Related Work

Currently, there are many network protocols for WSNs with their own advantages and disadvantages. Energy-based routing protocols [6] can choose transmission paths based on available energy of WSN nodes or energy requirement of links of transmission paths. However, such energy routing algorithms need to know the network global information, and the energy constraints of sensor networks only allows nodes to obtain local information, so they are routing methods just in ideal situations.

A negotiation-based routing algorithm such as the sensor protocol for information via negotiation or SPIN [7] is a data-centric self-adaptive communication routing protocol. Its goal is to offset the deficiencies in the diffusion method of SPIN through the negotiation mechanism between nodes and the resource self-adaptive mechanism. Its shortcoming is that in the process of new data transmission it directly broadcasts ADV data packages to its neighbor nodes, and that all neighbor nodes are not willing to take the responsibility of forwarding new data due to their own energy. Therefore new data cannot be transferred, data blind spots will appear, and the information collection across the whole network will be affected.

The minimum transmission energy [8] (MTE) protocol is an improved traditional direct route algorithm. In the MTE, a node chooses the one closest to itself for routing forwarding. Its advantage is simplicity, low cost, and each node only needs to find the next hop node to the sink node, and then sends the data to it. The disadvantage is that sensor nodes near the sink node always takes the role of router, loads between nodes are not in balanced, sensor nodes close to the sink node may soon run out of energy and “die”, and the life cycle of the entire network is shortened.

The Directed Diffusion [9] protocol is a data-centric routing protocol, and features the introduction of a gradient to describe the possibility of network intermediate nodes continuing their search for matched data along the direction. The disadvantage is that there are no multiple routes to the sink node, and the routing robustness is not good enough.

The LEACH & LEACH_C protocols: LEACH is a cluster-based routing protocol, and uses the following technologies to achieve its energy-saving: 1) random, self-adaptive, self-organization clustering method; 2) local control of data transmission; 3) low-energy consumption of the MAC protocol; 4) information processing technology. The LEACH protocol occupies an important position in WSN routing protocols, and other cluster-based routing protocols have been developed from the LEACH one. Its proposer later improved it, proposing the LEACH_C [10] protocol. The main improvement was that during the clustering nodes no longer compete for cluster heads, but nodes first send their own data to the sink node, and then the sink node determines the location of cluster heads according to their location, energy and cycle. The advantage of doing so lies in that we get a reasonable distribution of clusters through a reasonable arrangement of cluster heads, reducing the energy consumption due to the non-ideal random location or numbers of clusters in the original LEACH algorithm. For now, the LEACH_C and LEACH protocols may be considered generally equivalent cluster routing protocols.

Nevertheless, the LEACH_C network protocol has its own inherent shortcomings. Because the number of nodes in WSNs is large, the density coverage is also high, and the data collected by a single node are inevitably highly related with those collected by the entire WSN, and what users need is not the data collected by all nodes (including redundant data), but rather a description of incidents—the situation of events taking place in observed regions through the analysis of the set of network.

Taking it into account that the LEACH protocol uses clusters, data sent from nodes is processed locally (compression, de-redundancy) inside clusters and sent to the sink node. This will reduce the dependence of useful information users need on information a certain node collects. It also provides evidence for using the shift rest of nodes to reduce the energy consumption, that is to say, during each working cycle it will not affect the whole system greatly when a small amount of nodes in the network node hibernate.

Based on the above studies, this paper selects part of low energy nodes through cyclical filters, and puts them into the hibernation state to reduce the system’s total energy consumption, to extend the lifetime of the who system. At the same time, it requires consideration of various factors such as energy saving, self-adaptive capability of the network, the accuracy of information in accordance with the characteristics of WSNs when designing WSN protocols.

### 2.1. Structure Analysis of the Traditional LEACH Protocol

The traditional LEACH routing protocol is a low energy-consumption self-adaptive hierarchical routing algorithm designed for WSNs by Heinzelman *et al.* [9,11]. Its basic idea is that cluster heads are selected randomly through cycles with equal probability, and integrate and send information collected by members inside clusters to sink nodes to reduce communication traffic and distribute the energy load of the whole network evenly to each sensor node, so as to meet the goal of reducing energy consumption and extending the overall survival time of the network.

**Figure 2.**
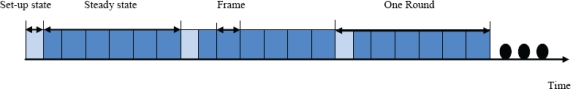
Working Cycle of the LEACH Protocol. Set-up state: selecting cluster heads, determining cluster members, and clusters coding and *etc.* Steady state: transferring data inside the temporary cluster structure.

The working process of the LEACH protocol is cyclic, defined as the concept of rounds. In Figure 2, each round of a cycle is divided into two types of state: set-up state and steady state.

In the set-up state, each sensor node competes for a cluster head at a certain probability and each cluster head broadcasts a message to all nodes that it has become one, and each node determines which one to join based on the signal strength, and responds to that cluster head. In the steady state, the cluster’s members send collected data to the cluster head according to the TDMA time slot, and the head integrates and sends data to sink nodes via Carrier Sense Multiple Access (CSMA). The steady state is much longer than the set-up state, and after a period of working time, the network re-enters a set-up state. The execution process is periodic. From the protocol process, cluster heads need to finish integrating data and communication with sink nodes, so the energy consumption is very high. The LEACH algorithm ensures that each node can be a cluster head in equiprobability, and that each node in the network consumes energy comparatively evenly.

### 2.2. Problem Description

During the operation process, the traditional LEACH protocol executes the cluster re-construction process constantly in cycles. The re-construction process is described in rounds, and each round needs to create clusters first. The process is mainly about how to select cluster heads. The protocol uses each sensor node to select a random number between 0∼1. If the selected number is less than T(n), then this node becomes a cluster head. This method of selecting cluster heads does not guarantee that cluster head nodes will be distributed over the entire network, and it is very possible for selected cluster heads to concentrate in a certain part of the network, resulting in no cluster heads around some nodes. The phenomenon of uneven distribution of cluster heads may result in premature death of nodes inside clusters, thus becoming blind nodes.

**Figure 3.**
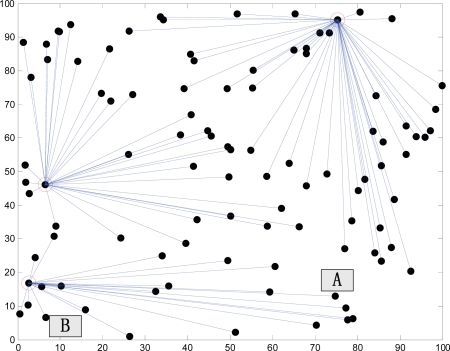
Diagrams for Reasons of Blind Nodes.

In Figure 3, nodes A and B belong to the same cluster. Suppose that *R_1_* is the distance from node A to the cluster head with energy *E_A_*, and *R_2_* is the distance from node B to the cluster head with energy *E_B_*, and that *E_A_* ≈ *E_B_*. *E_T_*, the communications energy, is proportional to the square of the distance to the target [8], and obviously *E_A_* − *E_TA_* < *E_B_* − *E_TB_*. The margin of the remaining energy increases as the margin between the distances from two nodes to the cluster head increases. If the location of the cluster head is selected improperly, resulting in that the margin between the distances to the cluster head is too large; the uneven level of energy of nodes inside the cluster may be very serious.

The traditional LEACH protocol assumes that all nodes carry the same energy. The selection of cluster heads is regardless to the remaining energy of nodes. However in the actual network operation, the remaining energy of nodes is often very different. It is necessary to take these factors into account for optimizing the selection of cluster heads.

## 3. Differential Evolution Algorithm

Because of the problems with the traditional LEACH protocol described above, this paper tries to use a more practical differential evolution algorithm to optimize the selection of cluster heads. The differential evolution (DE) algorithm is an emerging evolution algorithm, proposed by Storn *et al*. in 1995 [16,17]. The initial idea was to address the Chebyshev polynomial, and later it was found out that it was an effective technique for addressing complex optimization problems, and it has been successfully applied to solve the optimization of unconstrained single- and multi-objectives [18]. Like the traditional evolution algorithm, the particle swarm optimization (PSO) algorithm, *etc.*, the DE is an optimization algorithm based on the theory of swarm intelligence, and optimizes searches with swarm intelligence by cooperation and competition between individuals within the swarm.

**Figure 4.**
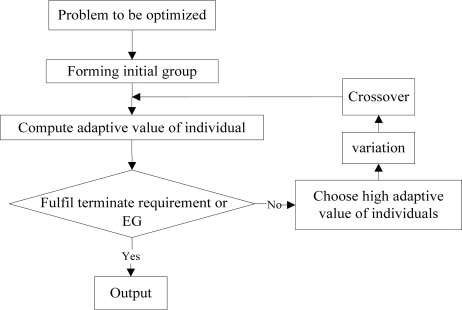
Flow Diagram of the Differential Evolution Algorithm.

Compared with the traditional evolution algorithms, the DE retains the swarm-based global search strategy, uses real-coded, simple compilation operations based on differences and the survival strategy of one-to-one competition, and reduces the complexity of genetic manipulation. In addition the DE dynamically tracks current searches with its unique memory capability to adjust its search strategy. With its comparatively strong global convergence capability and robustness and no need with the help from information about the characteristics of problems, it is applicable for complex optimization problems [19]. The basic flow is shown in Figure 4.

In the DE, there are similar operations such as mutation in genetic algorithms, crossover and selection. Among them, the mutation operation is defined as [18]:

(1) $$C={\text{Pr}}_{1}+F\;\left({\text{Pr}}_{2}-{\text{Pr}}_{3}\right)$$

where Pr_1_ Pr_2_ and Pr_3_, are three different individuals randomly selected from evolution swarm, and F is a parameter between the interval [0.5, 1]. In (1), the parameter *F* enlarges or shrinks the difference between two individuals, Pr_2_ and Pr_3_, randomly selected from the population swarm and adds them into the third individual Pr_1_, and a new individual *C*(*c*_1_, *c*_2_, ….,*c*_n_). To increase the diversity of swarms, z crossover operation is introduced into the differential algorithm, and the specific operation is as the follows: for each piece value *x_i_* of individuals Pr = (*x*_1_, *x*_2_,…,*x*_n_) of the father generation, a random pi is generated at the interval [0, 1]. The big or small relationship between Pr and the parameter CR determines whether *x_i_* is replaced with *c_i_* in order to obtain a new individual Pr′ = (*x*_1′_, *x*_2′_,…,*x*_n′_), in which xi′ = ci (when pi < CR) or xi (when pi ≥ CR). If the new individual Pr′ is better than the father-generation individual Pr, Pr will be replaced with Pr′. Otherwise it remains unchanged. In the differential evolution algorithm, selection operation uses the greedy strategy. Only when the generated offspring is better than father-generation individuals, the offspring will be retained. Otherwise, the father-generation individuals will be kept into the next generation.

## 4. LEACH Protocol Improvement Based on the Differential Evolution Algorithm

The LEACH protocol improvement based on the differential evolution algorithm executes in rounds the same as the traditional LEACH protocol. Each round execution is divided into four phases: (1) partitioning of initial clusters, (2) collecting status information about the nodes inside clusters by auxiliary cluster head nodes, (3) optimizing and selecting cluster heads with differential evolution algorithms, and (4) forming optimized clusters. Among them, the third one is the key to the improvements proposed in this paper, and will be described in detail.

### 4.1. Partitioning Initial Clusters

In this phase, the traditional LEACH routing algorithm is used for partitioning initial clusters for WSNs. The selection process of cluster head assignment by the LEACH protocol works as follows: each node creates a random number between 0∼1, and if the number is less than the threshold *T*(*n*), it sends a message to others that it is the cluster head. In each round of a cycle, if one node was a cluster head before, then *T*(*n*) is set to 0, so that node will not be elected again as a cluster head in this round. Non-elected nodes will be elected as cluster heads with probability *T*(*n*). The number of elected nodes increases, the threshold *T*(*n*) to elect the nodes from the remaining nodes increases, and the probability of the random number created by nodes less than *T*(*n*) increases, and the probability of nodes to become cluster heads increases correspondingly. When there is only one node that is not elected, *T*(*n*) = 1, which means the node must be elected as the cluster head. *T*(*n*) is given as:

(2) $$T(n)=\{\begin{array}{ll} \frac{P}{1-P\times [r\;\text{mod}(1/P)]}, & n\in G\\ 0, & \text{other} \end{array}$$

where *P* = *k/N* is the percentage of cluster head accounted for all nodes, r is the number of election rounds, r mod (1/*P*) refers to the number of nodes elected in the previous r-1 round of cycle, and G is a set of non-elected nodes in the previous r-1 round. It can be seen that the selection of cluster heads is determined by the elected times of nodes.

Nodes will broadcast ADV messages via the CSMA mechanism to all other ones to inform them that they became cluster heads when they compete successfully. The messages contain information such as IDs of cluster heads, *etc.* Non-cluster head nodes receive them, analyze and compare, find out the one with the strongest signal as its head cluster, and send a join-request message back to that cluster head, which contains the information of its own ID and that cluster head’s ID. When receiving it, that cluster head starts to create a TDMA schedule, uses the TDMA mechanism to allocate slots for each member in the cluster, and informs all nodes inside it. By now, the LEACH protocol has completed partitioning clusters. After that phase, cluster heads and clusters are basically determined, but blind nodes tend to appear in the clusters. Cluster heads in this phase is defined as auxiliary cluster heads.

### 4.2. Collecting Status Information of Nodes inside Clusters by Auxiliary Cluster Heads

Neighbor nodes inside the cluster send status information of their own locations and energy to auxiliary cluster heads. Now, the location *P*(*p*_1_, *p*_2_, ….,*p*_n_) and energy information *E*(*e*_1_, *e*_2_, ….,*e*_n_) of the neighbor nodes are stored in the auxiliary cluster heads, where n is the number of nodes in the cluster *p_i_* is the location value of node *i*, and *e_i_* is the energy value of node *i*.

### 4.3. Optimizing Algorithm and Determining Cluster Heads with the DE

The DE algorithm executes basically in the same way as other evolution algorithms, including coding, initial swarm formation, variation, crossover and selection operations. In order to make it suitable for the problem domain, the DE algorithm has to be modified, which is described in details as follows.

(1) Initial Swarm Formation: Initial swarms are formed with the corresponding groups of integer sequences of ID numbers of neighbor nodes the auxiliary cluster heads collect.

Defining the solution vector as:

(3) $$X_{i,G}=\left(x_{1},\;x_{2},...,\;x_{n}\right)\;\;i=1,\;2,\;...,\;\mathrm{NP}$$

Each solution vector is an evolution individual. Each generation of an evolution swarm is expressed as *G, i*, is an individual’s location in the swarm, *n* is the number of neighbor nodes auxiliary cluster heads collect, and *NP* is the scale of the swarm.

The first-generation swarm is created at random. The rule is to sort the IDs of neighbor nodes’ auxiliary cluster heads collected in an ascending order of *ID_1_* < *ID_2_* < …< *ID_n_*, to create a corresponding relationship of *ID_i_* ↔ *i*, *i* = 1,…, *n*, and to create the initial swarm based on that relationship. Each element in the swarm is given as:

(4) $$x_{\mathrm{ij},1}=1+\mathrm{round}\left[\mathrm{rd}\times \left(n-1\right)\right],\;\;i=1,2,\;...,\mathrm{NP}\;\;\;j=1,\;2,\;...,n.$$

where *rd* is a random number between (0,1), *round* [] is a closest integer, and *n* is the number of neighbor nodes.

(2) Variation Operation: Variation Operation is an important step for the DE algorithm to create sub-individuals. The algorithm uses the variation mode of DE/rand/1 [19]. Super-individuals, plus the difference between two or more individuals in the group create the sub-individuals. The basic unit of the variation operation is individual, which is solution vector.

For the evolution target vector of each generation *X_i,G_*, *i* = 1,2,…,*NP*, its variation operation is given by:

(5) $$V_{i,G+1}=X_{r_{1},G}+F(X_{r_{2},G}-X_{r_{3},G})$$

where *r*_1_, *r*_2_, *r*_3_ are not equal random integers in [1,2,*NP*], and not equal to *i. X_ri,G_* is called the father individual or basic individual. *F* is the variation factor, and *F* ∈ (0,1).

(3) Crossover Operation: The crossover operation adds varieties to the swarm. It includes two modes, index crossover mode and binomial crossover mode. The algorithm uses the common binomial crossover mode defined as

(6) $$u_{\mathrm{ji},G+1}=\left\{ \begin{array}{ll} v_{\mathrm{ji},G+1} & \mathrm{if}\;(\mathrm{randb}(j)\leq \mathrm{CR})\;\;\mathrm{or}\;\;j=\mathrm{randr}(i)\\ x_{\mathrm{ji},G} & \mathrm{if}\;(\mathrm{randb}(j)>\mathrm{CR})\;\;\mathrm{and}\;\;j\neq \mathrm{randr}(i) \end{array}\right.$$

where *randb*(*j*) is a random decimal figure between [0,1], *randr*(*i*) is a random integer between [1,*n*] and *CR* is the crossover factor.

(4) Selection Operation: In order to calculate the adaptation value of each experimental vector, it is necessary to establish an objective function first. Each vector is composed of n integers between 1∼*n*, corresponding to the ID numbers of the neighbor nodes, respectively. The purpose of establishing the function is to connect an actual physical relationship with abstract numbers in the algorithm to judge the quality of the vectors.

Determining the adaptation value of the experiment vectors not only needs to consider the energy of corresponding nodes, but also reflects the energy distribution of surrounding nodes. The further away from the node the neighbor nodes are, the greater the energy; and *vice versa*. Based on this characteristic, an objective function is defined as:

(7) $$f(i)=\frac{1}{n}\sum_{j=1}^{n}\left({\alpha e}_{j}+{\beta \bar{e}}_{j}\right)$$

where *α* + *β* = 1 and *α* ∈ [0,1], *β* ∈ [0,1]; *e_j_* is the energy of the corresponding node of element *j* in the experiment vector (node *j* in short); *ē_j_* is the average energy of nodes except node *j; α* is the influential energy factor of *e_j_*; and *β* is the influential energy factor of *ē_j_*. Adjusting *α* and *β* will adjust the contribution rate of *e_j_* and *ē_j_* to the adaptation value. In this paper, *α* = *β* = 0.5.

The average equivalent energy *ē_j_* is defined as:

(8) $${\bar{e}}_{j}=\frac{1}{n-1}\sum_{i=1,i\neq j}^{n}\;{\tilde{e}}_{i}$$

where *ẽ_i_* = *f*(*e_i_*, *r_i_*) is the equivalent energy of node *i*. Suppose *r_i_* is the distance from node *i* to node *j*, and *e_i_* is the remaining energy of node *i*. The remaining energy of node *i* and its distance to node *j* should be taken into account together for the structure of *f*(*e_i_,r*i). The following should be satisfied: the farther the distance from node *i* to node *j*, the less the equivalent energy of node *i*, and *vice versa*. The function reflects the characteristics of problems in simulation. In all, the target function is:

(9) $$f\;(i)=\frac{1}{n}\sum_{j=1}^{n}\left[{\alpha e}_{j}+\beta \frac{1}{n-1}\sum_{i=1,i\neq j}^{n}\;e_{i}\cdot \text{exp}(-\frac{r_{i}}{l})\right]$$

The experiment vector *U*_*i,G*+1_ resulted from the crossover operation is compared with the objective vector *X_i,G_* substituted into the vector objective function. If the objective function value of *U*_*i,G*+1_ is greater than that of *X_i,G_*, then *X_i,G_* is replaced with *U*_*i,G*+1_. And if not, *X_i,G_* will be kept, thus the next generation swarm is created. By now, a swarm has finished a generation of evolution. The DE algorithm repeatedly executes the steps of variation, crossover and selection until the termination condition is satisfied. The termination condition is usually the evolution generations, and the element with the absolute dominant amount position in the experiment vectors is just the corresponding number of the final cluster head.

### 4.4. Forming Optimized Clusters

In this phase, auxiliary cluster head nodes send the information of optimized cluster heads to nodes inside the cluster, Optimized cluster heads collect and integrate information of nodes inside the cluster. Forming optimized clusters comprehensively considers status information of neighbor nodes, so the energy loss of nodes inside the cluster is even to avoid frequent blind nodes.

## 5. Simulation Experiments

The open source network simulator NS2 developed by U.C. Berkeley for the simulation of various IP networks is used in this paper to simulate the improved routing algorithm, and to verify advantages of the improved LEACH protocol compared with simulation results of classical protocols such as the traditional LEACH protocol. The software was originally based on network design and simulation in UNIX systems. The simulation in this paper is conducted under the UNIX environment by CYGWIN in Windows XP, and on the basis of NS-2.27 simulation experiments are conducted. The complete simulation platform was WindowsXP+CYGWIN+NS-2.27.

### 5.1. Simulation Design

In this simulation experiment, 100 sensor nodes are distributed randomly in a simulation area of 100 m × 100 m. A sink node is at the location of coordinates (x = 50, y = 175). The bandwidth is set to 1 Mbps. The length of messages is 500 bytes. The head length of all messages is 25 bytes. The initial energy of each node is 2J and in isomorphism (the same performance).

In the simulation experiment, the wireless communications system model is composed of transmitting circuits, power amplifiers and receiving circuits. The power consumption of transmitting and receiving circuits is *E_elec_* = 50*nJ/bit*. When the sender transmits kbit to the receiver with the distanced, the power consumption of the sender and the receiver is the following respectively:

(10) $$E_{\mathrm{Tx}}(k,d)=E_{\mathrm{Tx-elec}}\;(k)+E_{\mathrm{Tx-amp}}\;(k,d)=E_{\mathrm{elec}}\;k+\varepsilon_{\mathrm{amp}}\;{\mathrm{kd}}^{n}$$

(11) $$E_{\mathrm{Rx}}\;(k)=E_{\mathrm{Rx-elec}}\;(k)=E_{\mathrm{elec}}\;k$$

where *n* is determined by the transmission path. If the transmission is in free space, *n* = 2. If inside clusters, *ε_amp_* *= 10 pJ/bit/m^2^*. If there exists multi-path fading, n = 4, cluster heads communicate with Sink nodes and *ε_amp_* = 0.0013 *pJ/bit/m^4^*.

### 5.2. Parameter Impact in the DE Algorithm

The parameters in the DE needing control include swarm scale *NP*, variation factor and crossover factor. Among them, *F* and *CR* have comparatively greater impact on the algorithm. In order to discuss the impact of those two key parameters on the algorithm, suppose that *NP* = 50 and remains unchanged. Select *CR* = 0.2, 0.4, 0.6, 0.8 and and F = 0.1, 0.2, 0.3 for crossover experiments, totally in 12 groups to record the evolution generations of the algorithm convergence. The convergence criteria is the absolutely dominant position of some element in the experiment vectors (90% of the total amount). Each group is experimented for five times, and the average values of the evolution generations of the convergence are selected. The experiment results are shown in Table 1.

The analysis of the simulation experiments shows that the impact of crossover factor and variation factor on the evolution process is interrelated. When the *CR* value increases gradually and *F* is selected differently from small to large values, the evolution speed differs more and more. Smaller F tends to premature while bigger F is in a slower evolution. Overall, as the crossover factor CR increases and the value of the variation factor F decreases, the evolution speed increases gradually. However, as the crossover factor CR the increases, the evolution process becomes more and more sensitive to the value of the variation factor F. Although the larger value of CR has a faster evolution speed, the probability of a local optimal solution and premature is also large. After repeated experiments, the authors propose the parameters CR = 0.6∼0.8 and F = 0.2∼0.3 for the conditions in the experiments.

### 5.3. Analysis

#### 5.3.1. Evaluation of Parameters

This paper mainly uses the following parameters to evaluate the advantages and disadvantages of a WSN.

(1) Self-Adaptive Capability of the Network: There are usually hundreds or thousands of nodes in sensor networks, and they are placed in remote and dangerous environment to meet users’ needs to extract information from the monitored regions. It requires nodes to be able to keep the communications between nodes without confirmed network components or without knowing the location of nodes in advance.

(2) Working Survival Period of the System: There is a serious energy constraint problem in sensor networks, so the primary design goal is to use the energy of sensor nodes with high efficiency to extend the survival time of sensor networks.

(3) Network Quality: Because adjacent nodes in WSNs have a strong data collection relationship, it will lead to the redundancy of data some nodes collect. WSNs are different from other wireless networks, and its users do not have access to data each node collect in the whole network, and care only about the description of events taking place in the monitored region by WSNs. Therefore, the quality of WSNS is not related with data a single node, but with a series of data the network collect.

#### 5.3.2. Analysis of Experiment Results

To test the advantages of the improved LEACH routing protocol algorithm based on the differential evolution algorithm (the DE_LEACH routing algorithm), this paper compares it with the traditional MTE protocol, the classical cluster-based routing algorithm LEACH protocol, the LEACH_C protocol in terms of network survival time and network quality. The network survival time is defined as the time needed from the start of the simulation to the end of last node consuming its energy. The network quality is defined as the differential rate of change of the total data amount sink nodes receive as time changes.

##### Comparison of WSN Survival Time

Figure 4 is a comparison chart of WSN lifetime. The abscissa is time, and the ordinate is the number of live nodes in the network. The DE_LEACH refers to the improved protocol. Figure 4 reflects the comparison of network survival time between the improved protocol and the traditional one. It can be seen that the improved the DE_LEACH routing algorithm proposed in this paper survives longer than the MTE protocol, the traditional LEACH, and the LEACH-C protocol algorithm.

Nodes die in less than 100 s because the energy consumption of nodes is not in balance in the MTE protocol. Nodes closer to sink nodes are always responsible for data relays, consume their energy very fast, and it affects the life cycle of the entire WSN. Comparatively speaking, the performance of the MTE protocol is the worst. The LEACH-C is also an improved LEACH protocol, and centrally distributes location, number and cluster size of cluster heads via sink nodes for more reasonable cluster partition. The LEACH-C protocol extends a life cycle longer than the LEACH protocol.

The LEACH protocol begins to fade in about 410 s. The DE_LEACH routing algorithm in this paper fades in about 520 s, and the time when the first node dies is 25% later. In addition, no node survives in the LEACH protocol in about 570 s, and that time in the DE_LEACH algorithm is about 740 s. After a period of its operation, the remaining energy of nodes in the network are not in balance in the traditional LEACH, and the DE_LEACH routing algorithm proposed in this paper can balance energy loss among nodes inside clusters to prevent premature blind nodes, thereby extending the survival time of the network. The larger the network scale, the more obvious the energy saving effect.

##### Comparison of WSN Quality

**Figure 5.**
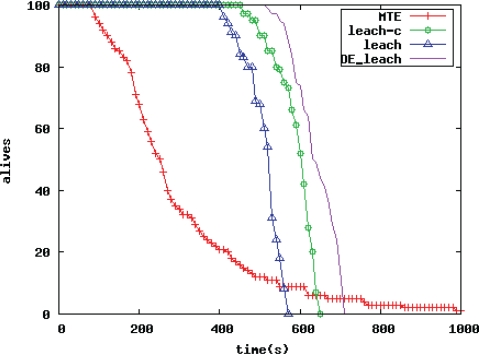
Comparison of WSN lifetime.

Figure 5 is a comparison chart of WSN quality. The abscissa is time, and the ordinate is the total data amount sink nodes receive. The chart reflects the network quality comparison between the improved DE_LEACH routing protocol and the traditional one.

In Figure 5, the network quality of the improved DE_LEACH routing algorithm is better than that of the MTE and the traditional LEACH protocol, and second only to the LEACH-C protocol. The MTE protocol has been at a comparatively lower level in terms of data amount, and the total data amount sink nodes receive is only about 11% of that the traditional LEACH protocol receives. In the traditional LEACH protocol, the total data amount sink nodes increases steadily before 400 s, and at about 580 s, the amount does not increase any more, meaning that the network life ends now. For the LEACH-C protocol, the cluster structure is more reasonable due to cluster heads allocated by sink nodes based on energy and location information, and the data amount is comparatively larger in the protocol, that is to say, a large data redundancy. But in about 650 s, the total data amount does not increase any more. In the proposed DE_LEACH protocol, the cluster distribution is more even, the number of cluster heads is more reasonable, and the data amount increases steady before 580 s until in about 700 s. Compared to the traditional LEACH protocol, the total data amount sink nodes receive increases around 35%. The larger the data amount the sink nodes receive, the greater the redundancy, the more accurate the judgment on the environmental monitoring.

Energy consumptions are usually compared with each other among those protocols that belong to the same category. In this paper, an improved version of the LEACH protocol is proposed. In comparison to the LEACH and the LEACH_C, since the number of clusters in the proposed protocol is more reasonable so that the speed of the energy consumption is much slower than that with LEACH and LEANCH_C, the time needed for the same energy consumption is thus much longer than them. Actually, our studies have shown that the survive time of the network nodes with the proposed protocol is more than those with other protocols, which is even more than 700 ms.

## 6. Conclusion

The ultimate goal of WSNs is to increase the sensing precision, accuracy of sensed information and to reduce redundant data of communications. The aim of precipitation monitoring, for example, is to improve the accuracy and real-time of precipitation data in the basin as well as to reduce the information loss of precipitation details. Existing hydrological stations must be built based on the conditions of good power supply, communications and transportation. The data from a single hydrographic station are often used to represent the precipitation information in its radius of a few hundred meters or even a few kilometers, and it would seriously affect the accuracy of regional precipitation information. WSNs for monitored environment avoid the dependence of monitor sites on the hydrological infrastructure, and geographical and terrain factors can be fully taken into account in the deployment of sensor nodes. The precipitation information collected via sensors can be representative for meteorological environment of the monitored region. In addition, the reduced cost of building stations and the reduced size of nodes make it possible to deploy more nodes. Based on requirements, the amount of precipitation information within the same area can be several, several hundred times or even higher than that of the existing hydrological station network. The decisions supported by that amount of information are much more accurate than by the traditional network.

In wireless sensor networks, the performance of routing protocols determines the overall performance of the network, and the WSN routing protocols have always been a hot topic. This paper takes outdoor environmental monitoring applications such as meteorology and hydrology and wetland ecology field as the background. The paper proposes an improved LEACH routing algorithm based on the differential evolution algorithm (DE_LEACH routing algorithm) to address the shortcomings of the traditional LEACH routing protocol. The improved algorithm uses the simple and fast search features of the differential evolution algorithm (DE) to optimize multiple objectives for the selection process of cluster heads to improve energy efficiency and stability of the application system. The simulation results prove that the DE_LEACH routing algorithm proposed in this paper can effectively prevent blind nodes in normal clustering routing algorithms, improve the life cycle of large-scale WSNs as well as the quality, and make WSN routing protocols with the improvement in this paper more suitable for outdoor environmental monitoring applications such as meteorology and hydrology, wetland ecology field than other routing protocols.

The present research results are mainly on how to avoid an unreasonable distribution of cluster heads and how to prevent the emergence of blind nodes. Further studies will focus on how to improve the selection for each parameter to adaptively meet the needs of various applications in order to reduce the computing amount as much as possible.

## Figures and Tables

**Figure 6.**
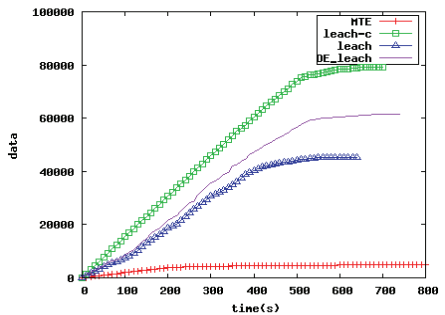
Comparisons of WSN Quality.

**Table 1.** Impact of Crossover and Variation Factors on the Evolution Generations.

*CR*	0.2			0.4			0.6			0.8		
*F*	0.1	0.2	0.3	0.1	0.2	0.3	0.1	0.2	0.3	0.1	0.2	0.3
Generations (average in five experiments)	543	557	621	115	208	327	102	165	246	46	103	154
